# The proteinuria selectivity index value predicts the remission of IgA nephropathy: a retrospective cohort study

**DOI:** 10.1080/0886022X.2024.2423839

**Published:** 2024-11-04

**Authors:** Ryunosuke Mitsuno, Takashin Nakayama, Wataru Ito, Tomomi Maruki, Ran Nakamichi, Keika Adachi, Norifumi Yoshimoto, Akihito Hishikawa, Aika Hagiwara, Shintaro Yamaguchi, Toshiaki Monkawa, Jun Yoshino, Akinori Hashiguchi, Tatsuhiko Azegami, Kaori Hayashi

**Affiliations:** a Division of Endocrinology, Metabolism and Nephrology, Department of Internal Medicine, Keio University School of Medicine, Tokyo, Japan; b Medical Education Center, Keio University School of Medicine, Tokyo, Japan; c Division of Nephrology, Department of Internal Medicine, and The Center for Integrated Kidney Research and Advance (IKRA), Faculty of Medicine, Shimane University, Shimane, Japan; d Department of Pathology, Keio University School of Medicine, Tokyo, Japan

**Keywords:** Biomarker, IgA nephropathy, proteinuria, proteinuria selectivity index

## Abstract

IgA nephropathy (IgAN) is the most common type of primary glomerulonephritis worldwide and leads to end-stage kidney disease. The proteinuria selectivity index (PSI) has been used to assess the prognosis in nephrotic syndrome, but its predictive value in patients with IgAN remains unclear. This single-center retrospective cohort study included patients who diagnosed with IgAN between March 2012 and March 2020. The PSI was calculated at the time of kidney biopsy. Patients were followed up from the time of kidney biopsy to kidney replacement therapy, death, transfer to another facility, or study completion. Ninety-four patients with a median age of 51 years were enrolled and divided according to the cutoff value of PSI determined by the receiver operating characteristic curve analysis into low-PSI (PSI <0.243, *n* = 39) and high-PSI groups (PSI ≥0.243, *n* = 55). The median follow-up duration was 70 months. Rates of remission of proteinuria and survival without a two-fold increase in serum creatinine were significantly better in the low-PSI group (both *p* < 0.01, log-rank test). Cox regression analysis showed that a low PSI was significantly associated with an increased likelihood of remission of proteinuria and hematuria (hazard ratio [HR] 1.96; 95% confidence interval [CI] 1.02–3.85 and HR 1.75; 95% CI 1.01–3.13, respectively), and a decreased risk of a two-fold increase in serum creatinine (HR 0.10; 95% CI 0.01–0.81). In conclusion, The PSI could have the potential to support the assessment of the prognosis of IgAN, in addition to established prognostic markers, by reflecting the overall glomerular permeability.

## Introduction

IgA nephropathy (IgAN) is the most common type of primary glomerulonephritis worldwide and is a leading cause of chronic kidney disease [1,2], ultimately leading to end-stage kidney disease in 30%–40% of patients after approximately 20 years [3]. IgAN also has a negative impact on life expectancy, reducing it by approximately 6 years [4]. Despite extensive research, IgAN remains a disease with many clinical challenges, including the development of an index that predicts the response to therapy.

Although the measurement of proteinuria is essential for predicting the prognosis of IgAN [2], standard tests cannot differentiate the size of protein in the urine. The proteinuria selectivity index (PSI) is the clearance ratio of a large protein (such as IgG) to that of a smaller protein (such as transferrin) [5] and can be used to assess changes in permeability of the glomerulus for large molecules in patients with glomerular disease. Injury to the glomerular barrier increases glomerular permeability, resulting in an elevated PSI or nonselective proteinuria. The PSI can predict the permeability of active nephron units and has been used widely to predict the efficacy of corticosteroids and the prognosis in nephrotic syndrome [6]. However, no definitive conclusion has been reached regarding the association between the PSI and the prognosis of IgAN. Therefore, the aim of this study was to determine whether PSI can predict the likelihood of remission of IgAN.

## Materials and methods

### Study population

The study had a single-center, retrospective, cohort design and was approved by the Keio University School of Medicine Ethics Committee (approval number: 20241041). Informed consent was obtained *via* the opt-out route. The study participants were patients aged ≥18 years who were diagnosed to have IgAN by kidney biopsy at our hospital between 1 March 2012 and 31 March 2020. Patients were excluded if they had missing PSI data, a baseline urine protein <0.3 g/day, or if they were not followed up in the outpatient setting after kidney biopsy owing to transfer to another facility.

The steroid treatment protocol was as described in the Japanese Society of Nephrology guidelines for IgAN [7]. Briefly, patients received methylprednisolone pulses of 500–1000 mg/day for three consecutive days and two additional pulses within the following 6 months. Prednisolone 0.5 mg/kg body weight was administered on alternate days during these 6 months and gradually tapered off within 2 months after the third pulse. The decision regarding administration of a renin-angiotensin system inhibitor (RASi), such as an angiotensin receptor blocker or angiotensin-converting enzyme inhibitor, and/or a sodium-glucose cotransporter 2 inhibitor (SGLT2i), as well as the indication for tonsillectomy, was left to the discretion of the attending physician.

### Follow-up

The participants were followed up from kidney biopsy until kidney replacement therapy, death, transfer to another facility, or study completion (31 April 2024).

The primary outcome was time to remission of proteinuria. The secondary endpoints were time to remission of hematuria and a two-fold increase in serum creatinine level, including initiation of kidney replacement therapy. Remission of proteinuria and hematuria was defined as disappearance of proteinuria and hematuria, respectively. Disappearance of proteinuria was defined as a urinary protein <0.3 g/day or g/g Cr or (−) to (±) on a dip-stick test for at least 6 months. Disappearance of hematuria was defined as <5 red blood cells in urine per high-powered field or (−) to (±) on a dip-stick test for at least 6 months. These definitions are based on the criteria proposed by the Japanese Society of Nephrology [8].

### Data collection

The following demographic data at the time of kidney biopsy were obtained from the electronic medical records: age, sex, body mass index, blood pressure (including use of antihypertensive medication), comorbidities, and treatment with RASi and SGLT2i before and after kidney biopsy, steroids, and tonsillectomy during the study period. The Charlson comorbidity index (CCI) was obtained from the medical records. The following biochemical data were also obtained: blood levels of albumin, creatinine, urea nitrogen, transferrin, IgG, IgA, complement C3, uric acid, total cholesterol, triglycerides, low-density lipoprotein cholesterol, HbA_1c_, and hemoglobin, urine levels of protein, albumin, creatinine, beta 2-microglobulin, transferrin, and IgG, and the red blood cell count in urine. The PSI was calculated as the clearance ratio of IgG to that of transferrin [5]. The estimated glomerular filtration rate (eGFR) was calculated using the three-variable Japanese equations, and the geriatric nutritional risk index was calculated using body mass index and the serum albumin level [9,10]. Pathology was graded according to the Oxford classification of IgA nephropathy [11], and the presence of cellular/fibrocellular crescents, percentage of global glomerulosclerosis in all glomeruli, percentage of interstitial fibrosis/tubular atrophy, interlobular artery intimal thickness, and arteriolar hyalinosis were assessed by pathologists from kidney biopsy samples.

### Statistical analysis

The study participants were divided according to the cutoff value of PSI determined by the receiver operating characteristic (ROC) curve analysis into a low-PSI group and a high-PSI group. Continuous variables are shown as the median (interquartile range [IQR]). Continuous variables were compared between the low-PSI and high-PSI groups using the Mann–Whitney *U* test. Categorical variables are shown as percentages and were compared between groups using Fisher’s exact test

Survival curves were plotted by the Kaplan–Meier method and compared between groups using the log-rank test. Cox proportional hazards models were used to determine hazard ratios (HRs) with 95% confidence intervals (CIs). In adjusted model 1, age and sex were included as candidate independent variables. The KDIGO guideline states that there are no validated prognostic serum or urine biomarkers for IgAN other than eGFR and proteinuria [2]. Additionally, the presence of crescents also significantly predicts outcome of IgAN [11]. Therefore, model 2 A included eGFR, urine protein, and presence of cellular/fibrocellular crescents as an independent covariate in addition to those included in model 1. The percentage of global glomerulosclerosis replaced eGFR in models 2B, and interstitial fibrosis/tubular atrophy replaced eGFR in model 2 C, and urine albumin-to-creatinine ratio replaced urine protein in model 2D; these variables were analyzed separately, taking multicollinearity into consideration. Model 3 included treatment with RASi and SGLT2i after kidney biopsy as well as the treatment protocol (with or without steroids and with or without tonsillectomy) as an independent covariate in addition to those in model 2 A. The same variables were also selected as potential covariates for multivariate analysis to assess their association with rates of remission of proteinuria and hematuria and the rate of survival without a two-fold increase in the serum creatinine level.

In sensitivity analysis, propensity score matching was used to assess the relationship between the PSI value and the likelihood of remission of proteinuria and hematuria, and the risk of a two-fold increase in the serum creatinine level. Propensity scores were calculated using a logistic regression model based on the following variables: age, sex, CCI, eGFR, urine protein, presence of cellular/fibrocellular crescents, treatment with RASi and SGLT2i after kidney biopsy, and the treatment protocol. The low-PSI and high-PSI groups were then matched 1:1 using a caliper width of 0.2 of the pooled standard deviation of the logit of the propensity score [12]. After matching, the survival curves were compared using the log-rank test. HRs with the 95% CIs were determined using a Cox proportional hazard model.

All statistical analyses were performed using EZR, a graphical user interface for R [13]. A two-tailed *P*-value <0.05 was considered statistically significant.

## Results

### Patient data

**Figure 1.**
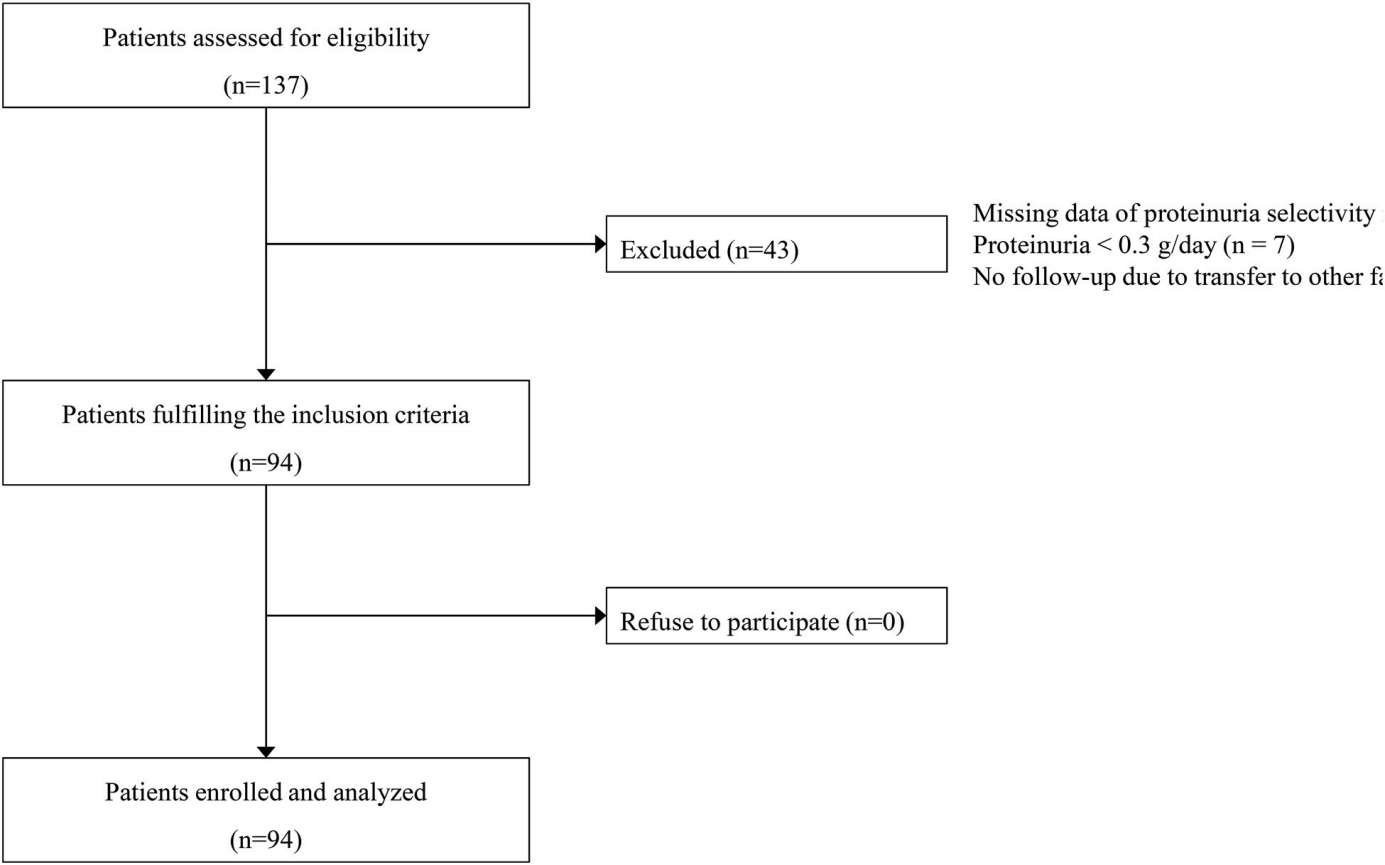
Flow chart showing the patient selection procedure.

Forty-three of the 137 enrolled patients were excluded because of missing PSI data (*n* = 34), proteinuria <0.3 g/day (*n* = 7), or loss to outpatient follow-up after kidney biopsy because of transfer to another facility (*n* = 2), leaving 94 patients for inclusion in the study (Figure 1). The median follow-up duration was 70 months (IQR 47, 102). Forty-eight patients (51%) achieved remission of proteinuria, 61 (65%) achieved remission of hematuria, and 22 (23%) experienced a two-fold increase in serum creatinine during the study period.

The ROC curve analysis was conducted to evaluate the predictive ability of PSI levels for the remission of proteinuria, showing an area under the curve of ROC (AUC) of 0.661 (95% CI 0.549–0.773), a cutoff value of 0.243, a sensitivity of 0.583, and a specificity of 0.783.

Table 1 summarizes the clinical characteristics for the total study group and when categorized according to whether the cutoff value of PSI determined by the ROC curve analysis was low (<0.243, *n* = 39) or high (≥0.243, *n* = 55). In the total study population, the median age was 51 years (IQR 35, 64), 43% were women, 54% had hypertension, and 10% had diabetes mellitus. Urine protein levels and urine albumin-to-creatinine ratio were significantly higher (*p* < 0.01 for both) and eGFR was significantly lower (*p* = 0.03) in the high-PSI group compared to the low-PSI group. There were no significant differences in the remaining variables or in the biochemical and pathological data. Among the 10 patients with negative hematuria at the time of renal biopsy, the duration of urine abnormalities was 10 years (IQR 2, 21), and their age was higher than that of the remaining 84 patients (60 years [IQR 51, 64] vs. 50 years [IQR 34, 63], *p* = 0.21). There were no significant differences in the prevalence of hypertension, interlobular artery intimal thickness, and arteriolar hyalinosis between the low-PSI and high-PSI groups.

**Table 1.** Characteristics of the study participants.

Variables	All patients			
	Total (*n* = 94)	Low PSI (*n* = 47)	High PSI (*n* = 47)	*P* value
Age (year)	51 [35, 64]	49 [32, 63]	54 [43, 64]	0.31
Sex (female)	40 (43)	20 (43)	20 (43)	>0.99
Hypertension	51 (54)	23 (49)	28 (60)	0.40
Diabetes mellitus	9 (10)	4 (9)	5 (11)	>0.99
Antihypertensive drugs before kidney biopsy				
RASi before kidney biopsy	37 (39)	14 (30)	23 (49)	0.09
CCB before kidney biopsy	32 (34)	14 (30)	18 (38)	0.51
SGLT2i before kidney biopsy	0 (0)	0 (0)	0 (0)	>0.99
Protein selectivity index	0.26 [0.21, 0.32]	0.21 [0.16, 0.23]	0.32 [0.30, 0.36]	<0.01
Charlson comorbidity index	0.00 [0.00, 1.00]	0.00 [0.00, 1.00]	0.00 [0.00, 1.00]	0.53
Systolic blood pressure (mmHg)	129.0 [117.0, 139.8]	128.0 [118.0, 137.5]	130.0 [114.5, 141.0]	0.33
Diastolic blood pressure (mmHg)	81.0 [73.0, 87.0]	80.0 [74.5, 85.5]	82.0 [70.3, 88.8]	0.86
Body weight (kg)	60.9 [54.5, 68.2]	61.0 [54.7, 67.8]	60.3 [53.9, 68.4]	0.97
Body mass index (kg/m^2^)	22.9 [20.4, 25.1]	22.9 [20.7, 24.9]	22.9 [20.5, 25.6]	0.65
Serum albumin (g/dL)	3.80 [3.40, 4.07]	3.80 [3.40, 4.00]	3.70 [3.30, 4.10]	0.90
Serum urea nitrogen (mg/dL)	18.5 [13.7, 23.0]	16.8 [13.9, 20.3]	20.6 [13.6, 26.6]	0.05
Serum creatinine (mg/dL)	1.17 [0.97, 1.56]	1.16 [0.90, 1.46]	1.28 [1.00, 1.71]	0.11
eGFR (mL/min/1.73m^2^)	45.0 [35.0, 65.0]	48.0 [38.0, 71.0]	43.0 [31.0, 60.5]	0.06
Serum IgA (mg/dL)	310.0 [234.8, 373.8]	303.0 [230.0, 390.0]	313.0 [260.0, 370.0]	0.67
Serum complement C3 (mg/dL)	104.0 [93.0, 120.0]	104.5 [95.3, 120.0]	102.0 [93.0, 119.0]	0.60
Urine protein (g/day)	1.23 [0.68, 2.17]	1.08 [0.65, 1.88]	1.65 [0.85, 2.83]	0.09
Urine albumin-to-creatinine ratio (mg/gCr) (*n* = 80)	660.8 [410.4, 1404.9]	528.4 [298.0, 877.7]	1013.1 [543.0, 1846.3]	<0.01
Urine RBC 0–4, 5–19, 20- (/HPF)	10 (11), 21 (22), 63 (67)	3 (6), 11 (23), 33 (70)	7 (15), 10 (21), 30 (64)	0.50
Urine beta 2-microglobulin-to-creatinine ratio (µg/gCr)	192.3 [103.2, 765.3]	171.6 [103.1, 382.5]	225.3 [116.8, 1198.3]	0.30
Serum uric acid (mg/dL)	6.50 [5.70, 7.45]	6.50 [5.75, 7.20]	6.50 [5.65, 7.85]	0.47
Serum total cholesterol (mg/dL)	209.0 [177.5, 234.8]	212.0 [184.5, 242.0]	207.0 [174.5, 232.5]	0.49
Serum triglycerides (mg/dL)	127.5 [95.0, 219.3]	140.0 [95.3, 220.8]	125.5 [96.3, 209.0]	0.97
Serum LDL cholesterol (mg/dL)	118.5 [98.3, 145.3]	121.0 [106.3, 148.3]	109.5 [86.8, 136.8]	0.12
Hemoglobin (g/dL)	12.9 [11.9, 14.2]	13.1 [12.1, 14.0]	12.8 [11.3, 14.5]	0.40
HbA1c (%)	5.50 [5.20, 5.73]	5.40 [5.12, 5.70]	5.60 [5.32, 5.80]	0.06
GNRI	99.1 [90.9, 108.1]	98.9 [91.6, 105.3]	99.9 [89.5, 108.7]	0.94
Oxford classification				
M 1 (*n* = 54)	2 (4)	2 (7)	0 (0)	0.49
E 1 (*n* = 54)	13 (24)	5 (18)	8 (31)	0.34
S 1 (*n* = 54)	40 (74)	20 (71)	20 (77)	0.76
T 1, 2 (*n* = 54)	15 (28), 5 (9)	10 (36), 1 (4)	5 (19), 4 (15)	0.19
Presence of cellular/ fibrocellular crescents	20 (21)	11 (23)	9 (19)	0.80
Global glomerulosclerosis (%)	23.1 [9.1, 43.8]	20.0 [6.8, 36.8]	26.2 [12.8, 50.0]	0.09
Interstitial fibrosis/ tubular atrophy 0–25, 26–50, 51– (%)	57 (61.3), 27 (29.0), 9 (9.7)	32 (69.6), 11 (23.9), 3 (6.5)	25 (53.2), 16 (34.0), 6 (12.8)	0.60
Interlobular artery intimal thickness no, mild/ moderate, severe (%)	27 (30), 55 (61), 8 (9)	14 (38), 21 (57), 2 (5)	13 (25), 34 (64), 6 (11)	0.32
Arteriolar hyalinosis no, mild/ moderate, severe (%)	35 (39), 53 (59), 2 (2)	17 (46), 20 (54), 0 (0)	18 (34), 33 (62), 2 (4)	0.38
Treatment				0.12
Non Steroid therapy	35 (37)	18 (38)	17 (36)	
Steroid without tonsillectomy	40 (43)	16 (34)	24 (51)	
Steroid with tonsillectomy	19 (20)	13 (28)	6 (13)	
Antihypertensive drugs after kidney biopsy				
RASi after kidney biopsy	64 (68)	30 (64)	34 (72)	0.50
CCB after kidney biopsy	34 (36)	17 (36)	17 (36)	>0.99
SGLT2i after kidney biopsy	30 (32)	13 (28)	17 (36)	0.50

Abbreviations: RASi, renin angiotensin system inhibitor; CCB, calcium channel blocker; SGLT2i, sodium-glucose cotransporter 2 inhibitor; eGFR, estimated glomerular filtration rate; RBC, red blood cells; LDL, low density lipoprotein; GNRI, geriatric nutritional risk index; M, mesangial hypercellularity; E, endocapilary hypercellularity; S, segmental glomerulosclerosis or adhesion; T, tubular atrophy/interstitial fibrosis. P value was obtained using the Mann–Whitney U test for continuous variables and Fisher exact test for proportions.

After propensity score matching, there were 28 matched pairs of patients in the low-PSI and high-PSI groups. There were no significant differences in any of the study variables or in the biochemical or pathological data between the two groups other than the PSI value (Supplementary Table 1).

### Association between PSI and remission of proteinuria

**Figure 2.**
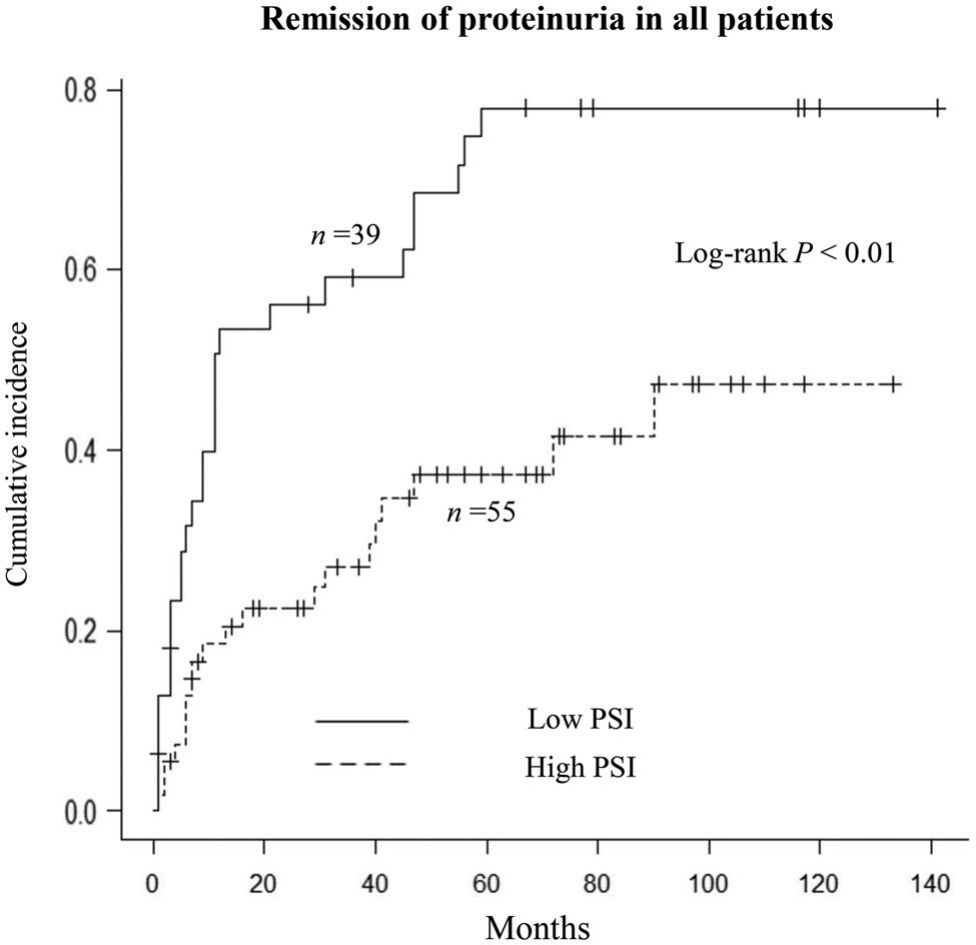
Kaplan–Meier analysis of time to remission of proteinuria in all patients according to whether the proteinuria selectivity index value was low or high.

During follow-up, remission of proteinuria was observed in 28 (72%) patients in the low-PSI group and 20 (36%) in the high-PSI group. The time to remission of proteinuria was significantly shorter in the low-PSI group than in the high-PSI group (11 months vs. not reached; *p* < 0.01; Figure 2). In multivariate analysis, a low PSI was independently associated with an increased likelihood of remission of proteinuria in model 3 (HR 1.96, 95% CI 1.02–3.85, *p* = 0.04; Table 2). The C-index value for model 3 was 0.75. After matching, the time to remission of proteinuria was significantly shorter in the low-PSI group than in the high-PSI group (31 months vs. not reached; *p* = 0.01; Supplementary Figure 1). A low PSI value was significantly associated with an increased rate of remission of proteinuria by Cox regression analysis (HR 2.66, 95% CI 1.20–5.91, *p* = 0.01).

**Table 2.** Association of protein selectivity index with remission of proteinuria and hematuria, and the incidence of two-fold increase in serum creatinine level and kidney replacement therapy using Cox regression analysis.

High PSI	Remission of proteinuria		Remission of hematuria		Two-fold increase in serum creatinine level	
	HR (95%CI)	P value	HR (95%CI)	P value	HR (95%CI)	P value
Cause-specific hazards						
Crude	2.63 (1.49–4.76)	<0.01	1.52 (0.91–2.50)	0.10	0.06 (0.01–0.46)	<0.01
Model 1	2.56 (1.45–4.55)	<0.01	1.69 (1.01–2.86)	0.04	0.06 (0.01–0.47)	<0.01
Model 2 A	2.04 (1.09–3.85)	0.02	1.92 (1.11–3.33)	0.01	0.09 (0.01–0.71)	0.02
Model 2B	2.04 (1.09–3.85)	0.02	2.00 (1.15–3.57)	0.01	0.08 (0.01–0.61)	0.01
Model 2 C	2.08 (1.10–4.00)	0.02	1.89 (1.06–3.33)	0.03	0.08 (0.01–0.62)	0.01
Model 2D	2.43 (1.20–4.88)	0.01	2.17 (1.18–3.85)	0.01	0.11 (0.01–0.84)	0.03
Model 3	1.96 (1.02–3.85)	0.04	1.75 (1.01–3.13)	0.04	0.10 (0.01–0.81)	0.03

Model 1 adjusted for age, sex. Model 2 A adjusted for the same variables as model 1 in addition to eGFR, urine protein, and presence of cellular/ fibrocellular crescents. Among the variables used in model 2 A, percentage of global glomerulosclerosis in all glomeruli is included instead of eGFR in model 2B, and interstitial fibrosis/ tubular atrophy is included instead of eGFR in model 2 C, and urine albumin-to-creatinine ratio is included instead of urine protein in model 2D. Model 3 adjusted for the same variables as model 2 A in addition to treatment with RASi and SGLT2i after kidney biopsy, and the protocol for treatment (with or without steroid, and with or without tonsillectomy). Abbreviations: HR, hazard ratio; CI, confidence interval; eGFR, estimated glomerular filtration rate; RASi, renin angiotensin system inhibitor; SGLT2i, sodium-glucose cotransporter 2 inhibitor.

### Association between PSI and remission of hematuria

**Figure 3.**
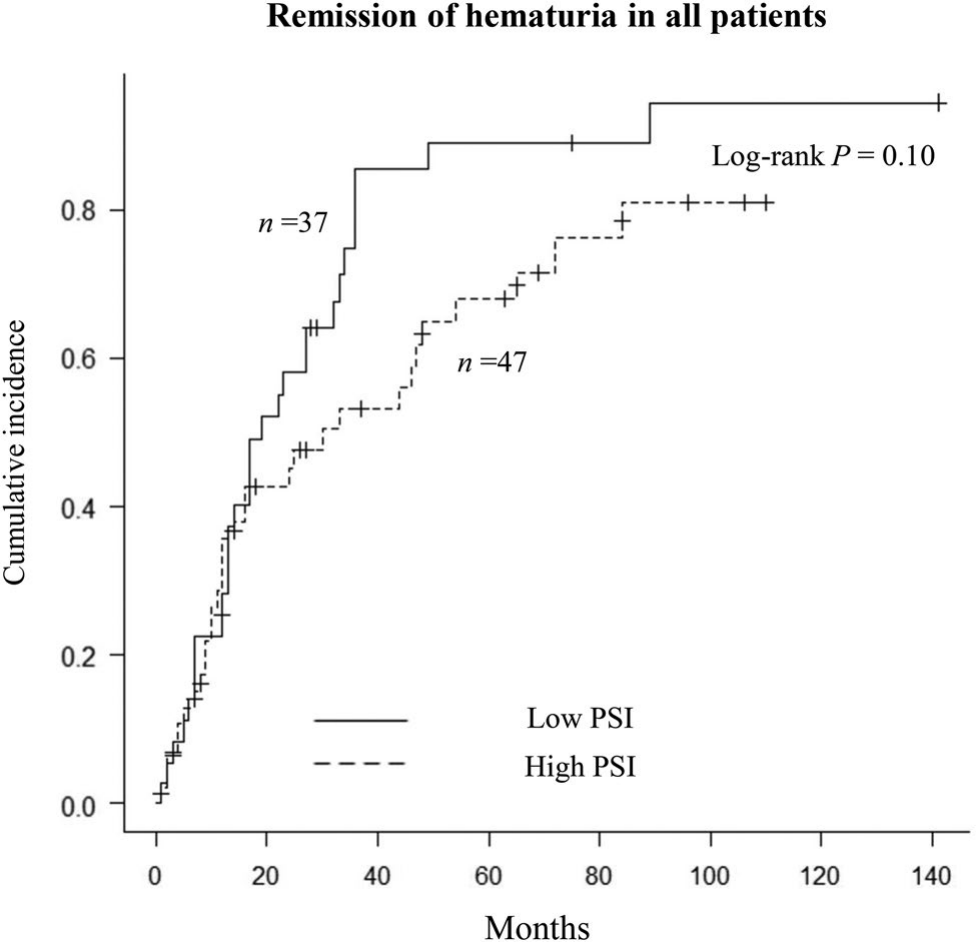
Kaplan–Meier analysis of time to remission of hematuria in all patients according to whether the proteinuria selectivity index value was low or high.

Two patients in the low-PSI group and 8 in the high-PSI group were excluded from this analysis owing to absence of hematuria at the time of kidney biopsy. Remission of hematuria was observed in 30 (81%) patients in the low-PSI group and 31 (66%) in the high-PSI group. Although there was no significant between-group difference in time to remission of hematuria, there was a trend toward this time being shorter in the low-PSI group than in the high-PSI group (19 months vs. 30 months; *p* = 0.10; Figure 3). Multivariate analysis using Cox regression showed that a low PSI was independently associated with an increased likelihood of remission of hematuria in model 3 (HR 1.75, 95% CI 1.01–3.13, *p* = 0.04; Table 2). The C-index value for model 3 was 0.72. After matching, the time to remission of hematuria was significantly shorter in the low-PSI group than in the high-PSI group (23 months vs. 54 months; *p* = 0.03; Supplementary Figure 2). A low PSI value was significantly associated with an increased rate of remission of hematuria by Cox regression analysis (HR 2.02, 95% CI 1.01–4.03, *p* = 0.04).

### Association between PSI and a two-fold increase in serum creatinine level

**Figure 4.**
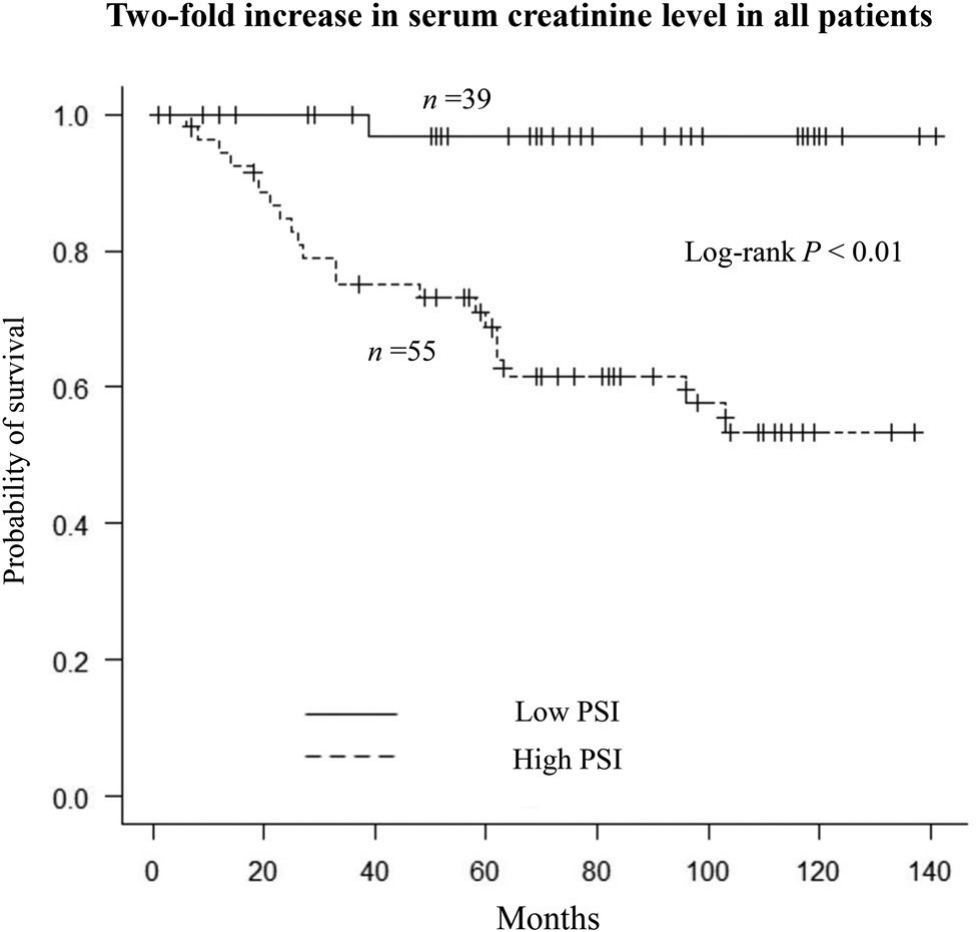
Kaplan–Meier analysis of survival time without a two-fold increase in the serum creatinine level in all patients according to whether the proteinuria selectivity index value was low or high.

A two-fold increase in the serum creatinine level was observed in 1 (2.6%) patient in the low-PSI group and 21 (38%) in the high-PSI group. Survival time without a two-fold increase in the serum creatinine level was significantly longer in the low-PSI group than in the high-PSI group (not reached vs. not reached; *p* < 0.01; Figure 4). Multivariate analysis using Cox regression revealed that a low PSI was significantly associated with a decreased risk of a two-fold increase in the serum creatinine level in model 3 (HR 0.10, 95% CI 0.01–0.81, *p* = 0.03; Table 2). The C-index value for model 3 was 0.91. After matching, Survival time without a two-fold increase in the serum creatinine level was significantly longer in the low-PSI group than in the high-PSI group (not reached vs. not reached; *p* = 0.01; Supplementary Figure 3). A low PSI value was significantly associated with a decreased risk of a two-fold increase in the serum creatinine level by Cox regression analysis (HR 0.13, 95% CI 0.02–0.99, *p* = 0.04).

## Discussion

There is a clinical need for an index that can predict the prognosis of IgAN. The findings of this retrospective cohort study provide evidence that a low PSI (<0.243) is associated with an increased likelihood of remission of proteinuria and hematuria, and a decreased risk of a two-fold increase in the serum creatinine level even after adjustment for potential confounders.

The PSI has been used widely to predict the efficacy of corticosteroids and the prognosis in patients with glomerular diseases [6]. However, there is limited information on the relationship between the PSI and IgAN. The first relevant research on this subject was an observational study published in 1986 by Woo et al. who found an association between nonselective proteinuria and glomerulosclerosis, hypertension, and a high serum creatinine level [14]. A further study by Woo et al. in 1989s investigated 98 patients with IgAN and found in univariate analysis that those with nonselective proteinuria had a significantly higher incidence of chronic kidney failure, defined as a serum creatinine level ≥1.6 mg/dL, during a 4-year follow-up period [15]. Although their research suggested that the PSI may be useful when predicting the prognosis of IgAN, there was a need for further studies.

To the best of our knowledge, this study is the first to evaluate the association between the PSI and the prognosis of IgAN, including remission of proteinuria and hematuria, by multivariate analysis with adjustment for baseline characteristics, namely, kidney function and current treatment, including steroids, tonsillectomy, and SGLT2i and RASi therapy. We have demonstrated a robust association of the PSI with remission of proteinuria or hematuria and two-fold increase in serum creatinine level even after adjusting for these potential confounders.

The mechanisms underlying our primary findings may be explained as follows. Several previous reports showed a correlation between the PSI and histological changes in the kidneys in patients with nephrotic syndrome and their response to steroid therapy [16–18]. The PSI aids in distinguishing focal segmental glomerulosclerosis from minimal change nephrotic syndrome by reflecting the presence of glomerulosclerosis lesions [6,19]. Moreover, Laurent et al. found that the correlation between PSI and responsiveness to steroids in patients with lipoid nephrosis was stronger than the correlation with the histopathological morphology obtained from kidney biopsy [20]. That finding reflects the difficulty in accurate evaluation of the true state of the kidney from samples containing only a small number of glomeruli and highlights the advantage of the PSI in reflecting the overall glomerular permeability. In the present study, after adjusting for potential confounders, a low PSI value was a significant predictor of remission of proteinuria and hematuria, even though no significant difference was observed in the available the Oxford pathological classification data between the low-PSI and high-PSI groups. Another perspective to consider is the possibility of nonselective protein leakage into the urine due to inflammation of the capillary walls. Since there was no significant difference in baseline urine red blood cell counts between the low-PSI and high-PSI groups, in cases with significant proteinuria but no hematuria, nonselective urinary protein leakage from sclerotic glomeruli could occur, suggesting that it may be difficult to assess the exact extent of capillary inflammation based solely on the degree of hematuria. Taken together, we speculate that focal segmental glomerular lesions, such as glomerulosclerosis, which could not be detected by kidney biopsy may cause the leakage of large protein molecules from the glomeruli in the high-PSI group in this study. Therefore, the PSI could have the potential to contribute to the current assessment of the prognosis of IgAN by reflecting the overall glomerular permeability.

Our study findings suggest that the PSI has clinical implications in patients with IgAN. There are two major challenges in terms of use of biomarkers in the treatment of IgAN, namely, establishing the prognosis and predicting treatment efficacy. IgAN remains a leading cause of chronic kidney disease in patients with primary glomerulonephritis [1]. However, predicting progression to kidney failure remains a major challenge for clinicians owing to the highly variable rate of decline in kidney function, even among patients diagnosed in the early stages of the disease [21]. In terms of predicting the efficacy of treatment, studies are ongoing to evaluate refined immunomodulation therapies, including drugs targeting the mucosal immune compartment, B cell-promoting cytokines, and the complement cascade in IgAN [22]. Given the rapid advances in therapeutics, the remaining gaps in biomarker research should be addressed. Currently, markers such as eGFR and proteinuria, along with histological scores, are used in the treatment selection and prognosis assessment of IgA nephropathy [2,21]. However, discrepancies between these markers are occasionally observed, underscoring the need for new markers to support prognosis prediction. From this perspective, PSI is a suitable marker that can reflect overall glomerular permeability, be performed simply and be repeated as necessary. We have demonstrated that the PSI can be used as a biomarker to predict the prognosis of IgAN. However, further studies are needed to evaluate its ability to predict the response to specific therapies.

Our study has some limitations. First, it had a retrospective observational cohort design that could not completely exclude the impact of all potential confounders, although we performed multivariate analyses. Due to the retrospective design, the direct causality between PSI and prognosis of IgAN is unclear. Second, the study was performed at a single center with a small sample size and had a short observation period, which limit its generalizability and statistical power. Nevertheless, the results of our sensitivity analyses were fairly consistent with our findings. Third, the Oxford pathological classification could not be included as a covariate in the analysis of this study. This limitation arose because, unfortunately, in some cases, not enough glomeruli were collected to evaluate the Oxford pathological classification, and there were considerable missing data on crescent scores (C0, C1, and C2) of MEST-C due to the inclusion of patients diagnosed with IgAN before the proposal of MEST-C. However, no significant difference in the available pathological staging was found between the low-PSI and high-PSI groups. Fourth, given the high median age of patients with IgAN in this study, we could not completely exclude the confounding effect of hypertensive nephrosclerosis with IgA deposition, although there were no significant differences in the prevalence of hypertension, interlobular artery intimal thickness, and arteriolar hyalinosis between the low-PSI and high-PSI groups. Finally, we did not evaluate the effects of the PSI on the ability to predict treatment efficacy. Of note, a recent study suggested a strong association between PSI and the prognosis in patients with primary nephrotic syndrome after treatment with rituximab [23]. Therefore, the PSI may serve as a predictive marker of the response to treatment and requires future investigation in patient with IgAN.

In conclusion, we have demonstrated that a low PSI value is significantly associated with an increased likelihood of remission of proteinuria and hematuria, as well as a decreased risk of a two-fold increase in the serum creatinine level. Our results suggest that the PSI could have the potential to support the assessment of the prognosis of IgAN, in addition to established prognostic markers, by reflecting the overall glomerular permeability. The relationship between the PSI and IgAN requires investigation in larger studies.

## Supplementary Material

sTables new.docx

## Data Availability

All data generated or analyzed during this study are included in this article and its supplementary material files. Further enquiries can be directed to the corresponding author.
