# Supplementary material for: The proteinuria selectivity index value predicts the remission of IgA nephropathy: a retrospective cohort study
Source: Ren Fail. 2024 Nov 4;46(2):2423839. doi: 10.1080/0886022X.2024.2423839 (PMC11536649; doi:10.1080/0886022X.2024.2423839)
Supplement: sTables new.docx [file IRNF_A_2423839_SM5121.docx]

| **Supplementary Table 1. Characteristics of the study in propensity-matched patients** | | | | | |
| --- | --- | --- | --- | --- | --- |
| Variables | | Propensity-matched patients | | | |
|  |  | Total (n=66) | Low PSI (n=33) | High PSI (n=33) | P value |
| Age (year) | | 50 [34, 64] | 50 [33, 67] | 50 [37, 62] | 0.74 |
| Sex (female) | | 29 (44) | 15 (46) | 14 (42) | >0.99 |
| Hypertension | | 38 (58) | 20 (61) | 18 (55) | 0.80 |
| Diabetes mellitus | | 7 (11) | 3 (9) | 4 (12) | >0.99 |
| Antihypertensive drugs before kidney biopsy | |  |  |  |  |
|  | RASi before kidney biopsy | 29 (44) | 12 (36) | 17 (52) | 0.32 |
|  | CCB before kidney biopsy | 24 (36) | 13 (39) | 11 (33) | 0.80 |
| SGLT2i before kidney biopsy | | 0 (0) | 0 (0) | 0 (0) | >0.99 |
| Protein selectivity index | | 0.26 [0.21, 0.32] | 0.21 [0.17, 0.23] | 0.32 [0.30, 0.36] | <0.01 |
| Charlson comorbidity index | | 0.00 [0.00, 1.00] | 0.00 [0.00, 2.00] | 0.00 [0.00, 1.00] | 0.58 |
| Systolic blood pressure (mmHg) | | 129.0 [117.0, 141.0] | 129.0 [118.0, 140.0] | 130.0 [115.0, 141.0] | 0.64 |
| Diastolic blood pressure (mmHg) | | 80.0 [73.0, 87.0] | 78.0 [73.0, 84.0] | 83.0 [74.8, 89.0] | 0.30 |
| Body weight (kg) | | 60.8 [53.4, 67.9] | 61.0 [54.5, 66.0] | 60.0 [52.9, 68.5] | 0.83 |
| Body mass index (kg/m^2^) | | 22.9 [20.1, 25.1] | 22.9 [20.0, 24.6] | 22.8 [20.2, 25.7] | 0.78 |
| Serum albumin (g/dL) | | 3.70 [3.32, 4.00] | 3.70 [3.40, 4.20] | 3.60 [3.20, 3.90] | 0.45 |
| Serum urea nitrogen (mg/dL) | | 18.7 [14.2, 23.1] | 18.6 [14.4, 22.4] | 18.7 [12.7, 25.9] | 0.69 |
| Serum creatinine (mg/dL) | | 1.23 [0.98, 1.63] | 1.17 [0.98, 1.48] | 1.23 [0.99, 1.70] | 0.58 |
| eGFR (mL/min/1.73m^2^) | | 43.5 [34.3, 63.0] | 44.0 [35.0, 65.0] | 43.0 [31.0, 63.0] | 0.62 |
| Serum IgA (mg/dL) | | 323.0 [237.3, 390.5] | 320.0 [224.0, 409.0] | 335.0 [270.0, 379.0] | 0.67 |
| Serum complement C3 (mg/dL) | | 103.0 [91.0, 119.0] | 102.0 [91.0, 110.8] | 107.0 [94.0, 120.0] | 0.49 |
| Urine protein (g/day) | | 1.59 [0.85, 2.85] | 1.17 [0.76, 2.14] | 1.83 [0.90, 2.90] | 0.25 |
| Urine albumin-to-creatinine ratio (mg/gCr) (n=47) | | 652.7 [407.9, 1040.8] | 645.1 [412.9, 979.4] | 779.6 [403.9, 1173.1] | 0.59 |
| Urine RBC 0-4, 5-19, 20- (/HPF) | | 6 (9), 16 (24), 44 (67) | 2 (6), 9 (27), 22 (67) | 4 (12), 7 (21), 22 (67) | 0.83 |
| Urine beta 2-microglobulin-to-creatinine ratio (µg/gCr) | | 180.0 [106.6, 398.3] | 210.6 [116.0, 552.9] | 177.5 [92.9, 307.7] | 0.41 |
| Serum uric acid (mg/dL) | | 6.60 [5.85, 7.57] | 6.50 [5.80, 7.30] | 6.60 [6.10, 7.80] | 0.29 |
| Serum total cholesterol (mg/dL) | | 211.0 [178.0, 232.5] | 212.0 [186.0, 231.0] | 206.5 [168.0, 233.8] | 0.51 |
| Serum triglycerides (mg/dL) | | 134.0 [89.5, 235.0] | 143.5 [89.5, 223.8] | 134.0 [96.8, 259.8] | 0.79 |
| Serum LDL cholesterol (mg/dL) | | 118.0 [96.5, 144.5] | 121.0 [103.8, 146.0] | 99.5 [75.8, 134.3] | 0.10 |
| Hemoglobin (g/dL) | | 12.7 [11.9, 14.1] | 12.7 [12.0, 14.0] | 12.7 [11.4, 14.2] | 0.63 |
| HbA1c (%) | | 5.60 [5.30, 5.80] | 5.45 [5.20, 5.70] | 5.60 [5.47, 5.82] | 0.10 |
| GNRI | | 97.7 [88.6, 108.1] | 98.1 [90.1, 105.3] | 97.2 [87.8, 108.7] | 0.89 |
| Oxford classification | |  |  |  |  |
|  | M 1 (n=38) | 1 (3) | 1 (5) | 0 (0) | >0.99 |
|  | E 1 (n=38) | 10 (28) | 4 (21) | 6 (35) | 0.46 |
|  | S 1 (n=38) | 28 (78) | 13 (68) | 15 (88) | 0.23 |
|  | T 1, 2 (n=38) | 11 (31), 3 (8) | 7 (37), 1 (5) | 4 (24), 2 (12) | 0.67 |
| Presence of cellular/ fibrocellular crescents | | 13 (20) | 5 (15) | 8 (24) | 0.53 |
| Global glomerulosclerosis (%) | | 26.2 [9.1, 44.4] | 25.5 [7.0, 39.0] | 26.7 [12.1, 50.0] | 0.33 |
| Interstitial fibrosis/ tubular atrophy  0-25, 26-50, 51- (%) | | 37 (57), 21 (32), 7 (11) | 21 (66), 8 (25), 3 (9) | 16 (49), 13 (39), 4 (12) | 0.36 |
| Interlobular artery intimal thickness no, mild/ moderate, severe (%) | | 17 (32), 31 (59), 5 (9) | 7 (27), 17 (65), 2 (8) | 10 (37), 14 (52), 3 (11) | 0.64 |
| Arteriolar hyalinosis no, mild/ moderate, severe (%) | | 22 (42), 29 (56), 1 (2) | 12 (46), 14 (54), 0 (0) | 10 (39), 15 (58), 1 (3.8) | 0.77 |
| Treatment | |  |  |  | 0.14 |
|  | Non Steroid therapy | 22 (33) | 14 (42) | 8 (24) |  |
|  | Steroid without tonsillectomy | 30 (46) | 11 (33) | 19 (58) |  |
|  | Steroid with tonsillectomy | 14 (21) | 8 (24) | 6 (18) |  |
| Antihypertensive drugs after kidney biopsy | |  |  |  |  |
|  | RASi after kidney biopsy | 49 (74) | 24 (73) | 25 (76) | >0.99 |
|  | CCB after kidney biopsy | 24 (36) | 13 (39) | 11 (33) | 0.79 |
| SGLT2i after kidney biopsy | | 24 (36) | 12 (36) | 12 (36) | >0.99 |
| Abbreviations: RASi, renin angiotensin system inhibitor; CCB, calcium channel blocker; SGLT2i, sodium-glucose cotransporter 2 inhibitor; eGFR, estimated glomerular filtration rate; RBC, red blood cells; LDL, low density lipoprotein; GNRI, geriatric nutritional risk index; M, mesangial hypercellularity; E, endocapilary hypercellularity; S, segmental glomerulosclerosis or adhesion; T, tubular atrophy/interstitial fibrosis. P value was obtained using the Mann–Whitney U test for continuous variables and Fisher exact test for proportions. | | | | | |
